# Effects of neutrophil granule proteins on sepsis-associated lymphopenia and their relationship with CD4^+^ T-cell pyroptosis

**DOI:** 10.3389/fimmu.2025.1507800

**Published:** 2025-02-07

**Authors:** Jia-yu Mao, Ya-wen Xie, Xian-li Lei, Jia-hui Zhang, Wei Cheng, Na Cui

**Affiliations:** Department of Critical Care Medicine, State Key Laboratory of Complex Severe and Rare Diseases, Peking Union Medical College Hospital, Chinese Academy of Medical Science and Peking Union Medical College, Beijing, China

**Keywords:** sepsis, sepsis-associated lymphopenia, neutrophil granule proteins, CD4 lymphocytes, pyroptosis

## Abstract

**Background:**

Neutrophil acts as a double-edged sword in the immune system. We hypothesized that an elevated neutrophil granule protein level is associated with sepsis-associated lymphopenia (SAL).

**Methods:**

We enrolled 61 patients with sepsis admitted to the Department of Critical Care Medicine of Peking Union Medical College Hospital between May 2022 and October 2023 in this study. Clinical and immunological parameters were recorded. Levels of neutrophil granule proteins, including myeloperoxidase (MPO) and neutrophil elastase (NE), and pyroptosis factors were examined.

**Results:**

Levels of neutrophil granule proteins (MPO, 82.9 vs. 175.3, *p* < 0 <.0001; NE, 56.3 vs. 144.2, *p* < 0.0001) were significantly higher in patients with sepsis with lymphopenia. Neutrophil granule protein levels were independently associated with SAL risk (MPO: OR = 1.0841, 95% CI, 1.0020–1.1730; NE: OR = 1.0540, 95% CI, 1.0040–1.1065). The area under the curve of MPO levels predicting SAL occurrence was 0.939 (95% CI, 0.846–0.984), and that of NE was 0.950 (95% CI, 0.862–0.989). Furthermore, neutrophil granule proteins were significantly correlated with CD4^+^ T cell and its pyroptosis [MPO and CD4^+^ T cells (*r* = −0.4039, *p* < 0.0001), CD4^+^NLRP3 (*r* = 0.4868, *p* < 0.0001), NE and CD4^+^ T cells (*r* = −0.5140, *p* < 0.0001), and CD4^+^NLRP3 (*r* = 0.6513, *p* < 0.0001)].

**Conclusion:**

Increased levels of neutrophil granule proteins were significantly associated with SAL incidence, and a significant relationship between neutrophil granule proteins and the pyroptosis pathway of CD4^+^ T cells was revealed.

**Clinical trial registration:**

chictr.org.cn identifier ChiCTR-ROC-17010750.

## Introduction

Sepsis is a life-threatening organ dysfunction caused by a dysregulated host response to infection (1). In this dysregulated response, many immune mechanisms that are initially activated to protect host are harmful, on account of both excessive inflammation and immune suppression (2). The mechanistic underpinnings of concurrent immune suppression and relevant immune system changes in patients with sepsis still need further exploration.

Polymorphonuclear neutrophils are traditionally regarded as professional phagocytic and acute inflammatory cells that engulf the microbial pathogens (3). As an innate immune system response, antigen presentation triggers the adaptive immune system to activate cytotoxic T lymphocytes and induce B lymphocytes to secrete specific antibodies. Upon activation, neutrophils extruded their nuclear DNA and histones in association with the attached granule proteins to form neutrophil extracellular traps (NETs) to trap the environmental foreign invaders (4). However, granule proteins not only contribute to killing bacteria within the phagosome, but also are capable of inflicting tissue damage (5), which prompted us to explore the interaction of neutrophil granule proteins with adaptive immune cells and their contribution to sepsis-associated immunosuppression.

Pyroptosis is a type of programmed cell death mediated by caspase-dependent activation of members of the gasdermin family and inflammasome formation (6). In patients with sepsis, pyroptosis is observed in multiple immune cells (7). Our previous study revealed that a reduction in CD4^+^ T cells is central to the immunosuppression phase of sepsis and that the canonical pyroptosis pathway obliterates CD4^+^ T cells (8). NETs have already been verified to interact with the NLRP3 inflammasome in septic lung injury (9, 10). In this research, we aimed to explore the relationship between neutrophil granule proteins and sepsis-associated lymphopenia (SAL) and its interaction with CD4^+^ T-cell pyroptosis. We hypothesized that the level of neutrophil granule proteins may reflect the occurrence of SAL through the pyroptosis pathway of CD4^+^ T cells.

## Materials and methods

### Patients and study design

A prospective study was performed from May 2022 to October 2023 in the Department of Critical Care Medicine of Peking Union Medical College Hospital (PUMCH). The PUMCH institutional Ethic Committee approved this study (approval number JS-1170), and all patients enrolled in this study signed informed consent. The study was registered at the Chinese Clinical Trial Registry (ChiCTR-ROC-17010750).

The inclusion criteria were as follows: (1) ≥ 18 years old, (2) ICU (intensive care unit) stay time over 1 day, and (3) diagnosis of sepsis (see below). Patients had basic immunological diseases or malignant tumors were excluded. Sepsis was diagnosed according to the definition of the Third International Consensus (1); lymphopenia was diagnosed if the total lymphocyte count was < 1.1 × 10^9^/L according to the 2022 SAI expert consensus (11).

### Outcome

Basic clinical evaluation and laboratory characteristics were collected on the first day of ICU admission. Basic clinical data, including age, sex, and basic information on immunological diseases and malignant tumors, were collected. Laboratory characteristics such as routine blood biochemistry, immunological parameters such as the peripheral blood lymphocyte count and lymphocyte subset counts, the SOFA (Sequential Organ Failure Assessment) score, and the APACHE (Acute Physiology and Chronic Health Evaluation) II score were recorded. Information on life-sustaining treatment, including the need for mechanical ventilation and renal replacement, was recorded. The follow-up data were 28-day mortality rate and ICU stay time.

Neutrophil granules can be divided into four groups according to the different maturation stages (12), and the levels of myeloperoxidase (MPO) and neutrophil elastase (NE) were measured as primary or azurophil granules. The NOD-like receptor family pyrin domain containing 3 (NLRP3) is a type of cytoplasmic pattern recognition receptor that may trigger inflammasome formation. NLRP3 was measured to evaluate the inflammasome and quantify pyroptosis in cells (13, 14). As the upstream canonical activation pathway of pyroptosis, the levels of NLRP3, Caspase-1 and gasdermin D (GSDMD) were measured gasdermin D (GSDMD) were measured (15).

**Figure 1:**
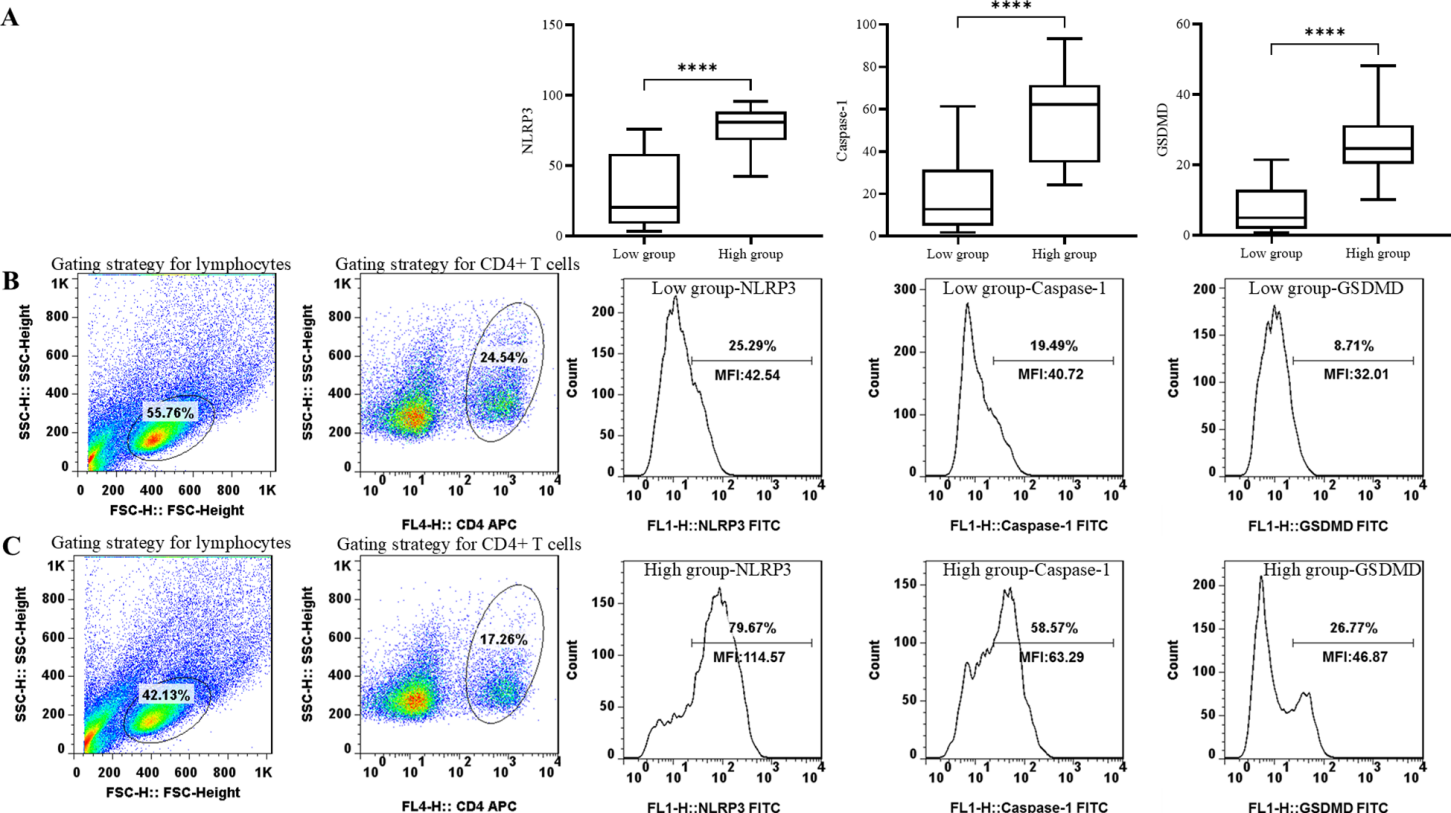
Comparison of pyroptosis biomarker levels, including NLRP3, Caspase-1, and GSDMD in groups with different neutrophil granule protein levels. ****p < 0.0001 **(A)** Comparison of percentage of NLRP3^+^ CD4^+^ T cells, percentage of Caspase-1^+^ CD4^+^ T cells, and percentage of GSDMD^+^ CD4^+^ T cells between patients with low neutrophil granule protein level and high neutrophil granule protein level. **(B)** Representative flow dot plots of the lymphocyte gating strategy of the lymphocyte, CD4^+^ T cell, NLRP3 MFI on CD4^+^ T cells, Caspase-1 MFI on CD4^+^ T cells, and GSDMD MFI on CD4^+^ T cells in patients with low neutrophil granule protein level. **(C)** Representative flow dot plots of the lymphocyte gating strategy of the lymphocyte, CD4^+^ T cell, NLRP3 MFI on CD4^+^ T cells, Caspase-1 MFI on CD4^+^ T cells, and GSDMD MFI on CD4^+^ T cells in patients with high neutrophil granule protein level.

Within 24 h of ICU admission, peripheral blood samples were collected. Blood sample was centrifuged immediately, and plasma was collected and stored at −80°C. Peripheral blood mononuclear cells (PBMCs) were purified by Ficoll density-gradient separation. Plasma and PBMCs were stored in the Clinical Biobank (ISO 20387: 2018) of Peking Union Medical College Hospital. The levels of MPO and NE were quantified by enzyme-linked immunosorbent assay (ELISA) (MPO: JL11580-96T Beijing China; NE: JL12352-96T Beijing China). For phenotypic staining, antibodies specific to CD4 (BioLegend, 980802, San Diego, CA), NLRP3 (Invitrogen, PA5-79740, Thermo Fisher Scientific, Inc.), Caspase-1 (Invitrogen, PA5-140994, Thermo Fisher Scientific, Inc.), and GSDMD (Invitrogen, PA5-119680, Thermo Fisher Scientific, Inc.) were used for surface staining of cells. Thawed PBMCs were resuspended and incubated with antibody under absolute dark conditions in room temperature. Having been washed by staining buffer, cells were fixed and permeabilized according to the instructions. The gating strategy of flow cytometry experiments is shown in **Figure 1**. Lymphocytes were gated on side scatter (SSC) and forward scatter (FSC). T cells (CD3^+^), CD4^+^, and CD8^+^ T-cell subsets were quantitated. Then, CD4^+^ lymphocytes were gated and immunoassayed with combinations of fluorochrome-conjugated antibodies, including NLRP3, Caspase-1, and GSDMD. The percentages of CD4^+^ lymphocytes expressing NLRP3, Caspase-1, and GSDMD and the MFI of these markers on CD4^+^ lymphocytes were further quantitated. A total of 10,000 cells were analyzed from each sample. The samples were analyzed through a BDCalibur flow cytometer (BD Biosciences) with FlowJo software (BD Biosciences).

### Statistical analysis

Normally distributed data are shown as the mean with standard deviation and were analyzed with Student’s *t*-test. Nonnormally distributed data are expressed as the median and interquartile range and were compared through the Mann–Whitney *U* test. Categorical variables are recorded as proportions and were analyzed by the chi-square test. Logistic regression was performed to identify parameters with predictive value for SAL risk. Statistically significant variables were subsequently analyzed using a multi-logistic regression, and the results were expressed as *P* and odds ratio (OR) with 95% confidence interval (CI). The value of neutrophil granule proteins for SAL risk prediction was calculated via ROC (receiver operating characteristic) curve analysis with the Hanley–McNeil test. The area under the curves (AUCs) were examined to determine a cutoff level to predict SAL occurrence. Based on the cutoff level, all the patients were divided into a low neutrophil granule protein group and a high neutrophil granule protein group. Pearson’s correlation analysis was performed for the detection of correlation between neutrophil granule proteins and expression levels of pyroptosis markers on CD4^+^ T cells. Statistical analyses were performed with the SPSS 13.0 software package (SPSS, Chicago, USA).

## Results

### Basic characteristics of study population in SAL and non-SAL groups

**Figure 2:**
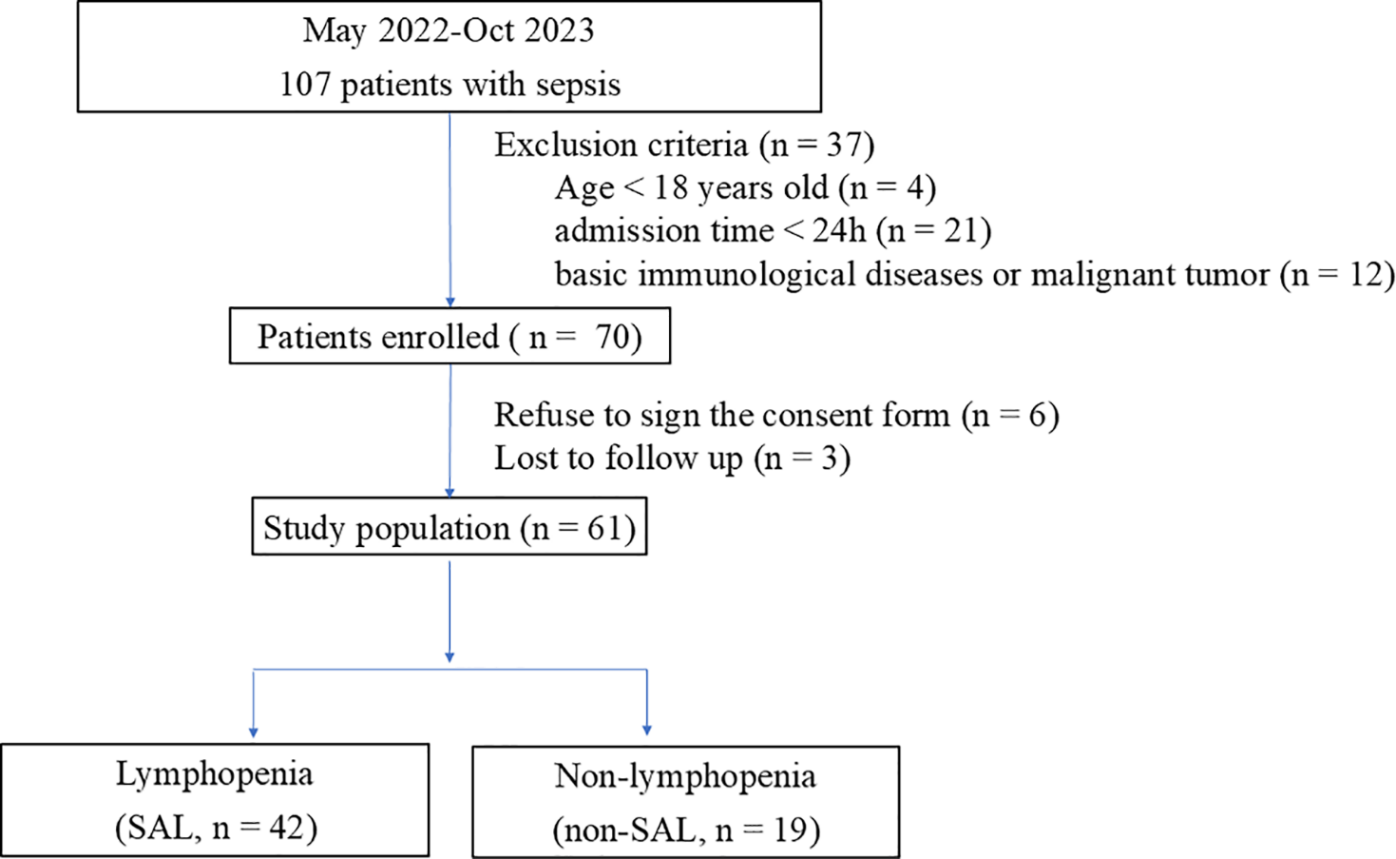
Flowchart of patients included in the study.

From May 2022 to October 2023, 107 patients admitted to the Critical Care Medicine Department of PUMCH were diagnosed with sepsis. Among these patients, 25 who did not meet the inclusion criteria (4 were < 18 years old and 21 were discharged within 24 h) were excluded, 12 patients had basic immunological diseases or malignant tumors, 6 refused to sign the informed consent, and 3 had withdrawn. A total of 61 patients were enrolled in this research, 42 of whom met the criteria for lymphopenia (**Figure 2**).

Basic clinical characteristics after ICU admission were compared between patients with or without SAL (non-SAL group vs. SAL group), as shown in **Table 1**. Age of patients between the different groups was accordant, and the majority of patients were male. Infection sources were similar in patients with SAL and those without SAL. The level of C-reactive protein in the SAL group (111.1 vs. 172.3 mg/L, *p* = 0.0371), but not the level of procalcitonin, was significantly greater. The mortality (0 vs. 19%, *p* = 0.0488), ICU stay time (2 vs. 7 days, *p* = 0.0176), SOFA score (6.3 vs. 9.0, *p* = 0.0201), APACHE II score (11.9 vs. 17.5, *p* = 0.0176), and need for mechanical ventilation (68.4 vs. 92.3%, *p* = 0.0207) and renal replacement (5.3 vs. 28.6%, *p* = 0.0474) were significantly greater in the SAL group than in the control group. No significant difference in white blood cell count was shown between the groups.

**Table 1:** Basic characteristics of Study Population in SAL and Non-SAL Groups included in this study.

Characteristics	Non-SAL, n = 19	SAL, n = 42	p
Age, years	61.6 (48.2 – 74.9)	60.8 (43.4 – 78.2)	0.8708
Sex, n (%) Male	13 (68.4)	26 (61.9)	0.7754
Infection site, n (%)			
Bloodstream	3 (15.8)	6 (14.3)	0.9999
Lung	10 (52.6)	16 (38)	0.5792
Abdominal cavity	7 (36.8)	20 (45.2)	0.2763
Soft tissue	3 (15.8)	2 (4.8)	0.1696
Pathogen, n (%)			
Gram-negative bacilus	9 (47.4)	27 (64.3)	0.2659
Gram-positive coccus	6 (31.6)	19 (50)	0.5792
Gram-positive bacilus	2 (10.5)	2 (4.8)	0.5820
Fungus	3 (15.8)	12 (28.6)	0.3507
PCT, ng/mL	0.5 (0.2 – 2.0)	5.4 (0.8 – 11.0)	0.0964
CRP, mg/L	111.1 (32.4 – 189.9)	172.3 (61.3 -283.4)	0.0371^*^
28-day mortality, n (%)	0 (0)	8 (19)	0.0488^*^
ICU stay time, days	2.0 (2.0 – 4.0)	7.0 (3.0 – 9.8)	0.0176^*^
APACHE II	11.9 (5.3 – 18.5)	17.5 (11.1 – 23.9)	0.0176^*^
SOFA	6.3 (2.4 – 10.2)	9.0 (5.0 – 12.9)	0.0201^*^
MV, n (%)	13 (68.4)	39 (92.3)	0.0207^*^
CRRT, n (%)	1 (5.3)	12 (28.6)	0.0474^*^
Creatinine, umol/L	72 (51 – 106.5)	76.5 (56.5 – 109.8)	0.5998
TBil, umol/L	13.1 (9.7 – 18.7)	16.0 (10.4 – 34.8)	0.2334
WBC, *10^9/L	14.8 (7.0 – 22.7)	13.0 (6.5 – 19.5)	0.3526
Neutrophil#, *10^6/L	12.9 (5.7 – 20.1)	11.9 (6.0 – 17.9)	0.5846
LY#, *10^6/L	1568 (930 – 2208)	572 (331 – 812)	<0.0001^****^
CD4^+^ T#, *10^6/L	544 (349 – 739)	218 (81 – 355)	<0.0001^****^
CD8^+^ T#, *10^6/L	411 (92 – 731)	139 (54 – 224)	<0.0001^****^
IL-6, pg/mL	78.2 (22.9 – 113.0)	128.7 (36.9 – 559.1)	0.1129
IL-8, pg/mL	45.5 (31.0 – 69.9)	48.9 (28.9 – 135.3)	0.2271
IL-10, pg/mL	6.5 (5.0 – 19.3)	10.8 (5.2 – 19.7)	0.5418
TNFα, pg/mL	11.1 (2.5 – 19.8)	11.2 (2.5 – 18.6)	0.5057
MPO, ng/mL	82.9 (67.6 – 98.1)	176.2 (99.6 – 252.9)	<0.0001^****^
NE, ng/mL	56.3 (35.9 – 76.6)	144.2 (86.9 – 201.6)	<0.0001^****^

*p<0.05; ****p<0.0001.

**Figure 3:**
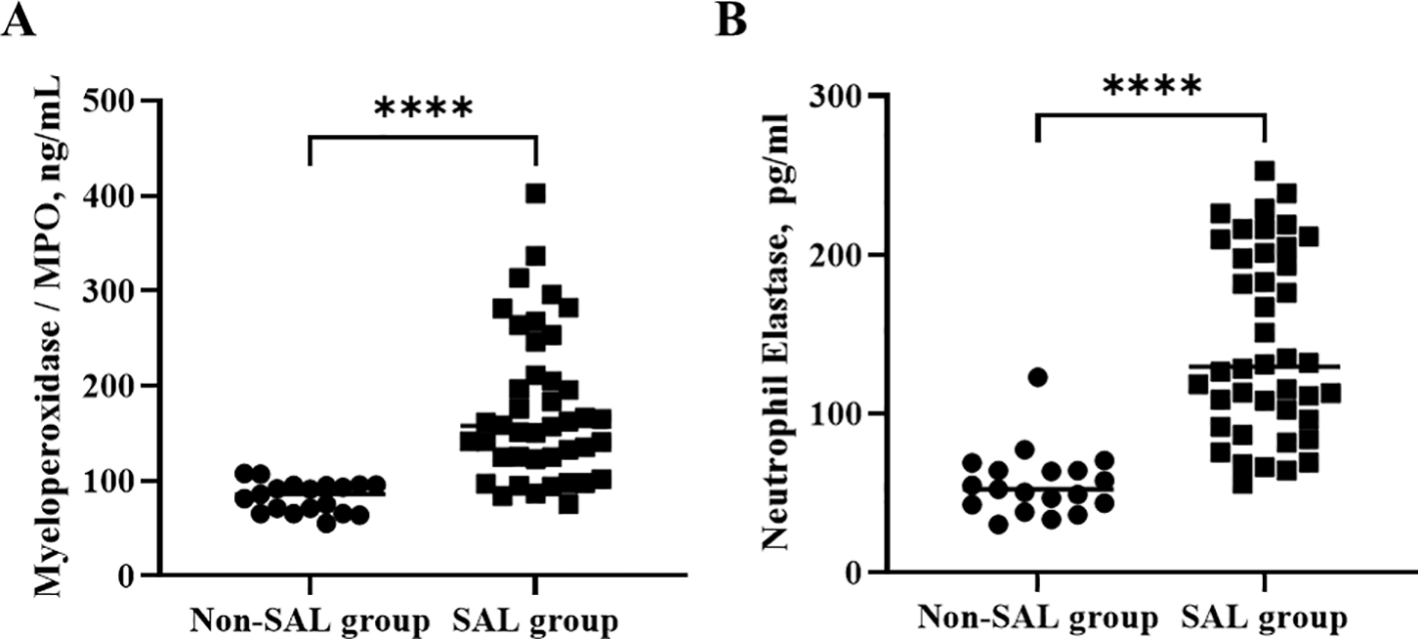
Comparison of neutrophil granule protein levels, including MPO **(A)** and NE **(B)** in groups of patients with sepsis with or without lymphopenia. ****p < 0.0001. SAL, sepsis-associated lymphopenia.

In terms of the lymphocyte counts and their subsets, the lymphocyte count (1,568 vs. 572 × 10^6^/L, *p* < 0.0001) and CD4^+^ T-cell (544 vs. 218 × 10^6^/L, *p* < 0.0001) and CD8^+^ T-cell counts (411 vs. 139 × 10^6^/L, *p* < 0.0001) were significantly lower in the SAL group. The levels of neutrophil granule proteins, including MPO (82.9 vs. 175.3, *p* < 0 <.0001) and NE (56.3 vs. 144.2, *p* < 0 <.0001), were significantly higher in the SAL group, as shown in **Figure 3**. In summary, patients with SAL showed significantly higher levels of neutrophil granule proteins.

### Neutrophil granule protein levels are a risk factor for lymphopenia in patients with sepsis

**Figure 4:**
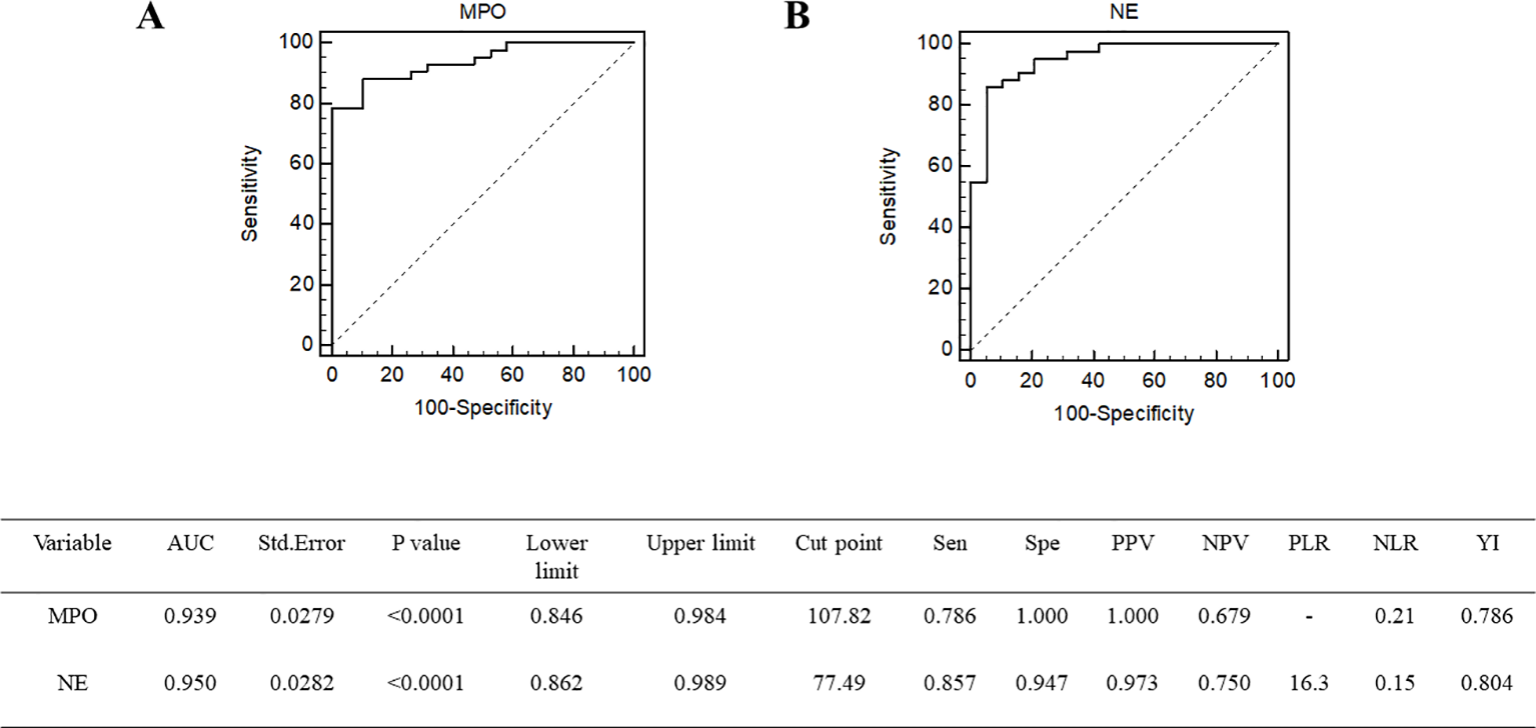
Receiver operating characteristic curve of MPO **(A)** and NE **(B)** for the prediction of SAL occurrence. MPO, myeloperoxidase; NE, neutrophil elastase.

Lymphopenia risk in patients with sepsis was examined by univariate logistic regression analysis (**Table 2**). Among them, the variables SOFA score, APACHE II score, MPO, and NE were associated with lymphopenia risk in patients with sepsis. After further adjustment by multivariate logistic regression analysis, the OR of MPO after adjustment was 1.0841 (95% CI, 1.0020–1.1730, *p* = 0.0446), and the OR of NE after adjustment was 1.0540 (95% CI, 1.0040–1.1065, *p* = 0.0339) (**Table 3**). To further determine the value of neutrophil granule proteins for SAL risk prediction in patients with sepsis, ROC curve analysis was generated. The area under the ROC curve for the prediction of SAL occurrence of MPO was 0.939 (95% CI, 0.846–0.984; *p* < 0.0001), and that of NE was 0.950 (95% CI, 0.862–0.989; *p* < 0.0001) (**Figure 4**).

**Table 2:** Univariate logistic regression analysis for possible risk factors for SAL.

					95% CI for OR	
Variable	B	SE	p	OR	Lower	Upper
Age	0.0029	0.0164	0.8617	1.0029	0.9712	1.0356
APACHE II	0.1384	0.0520	0.0077	1.1485	1.0373	1.2716
SOFA	0.1532	0.0732	0.0363	1.1656	1.0099	1.3453
MPO	0.0862	0.0279	0.0020	1.0900	1.0320	1.1513
NE	0.0792	0.0238	0.0009	1.0824	1.0331	1.1341

**Table 3:** Multivariate logistic regression analysis of possible risk factors significantly in predicting SAL in Sepsis Patients.

					95% CI for OR	
Variable	B	SE	p	OR	Lower	Upper
APACHE II	0.0568	0.1035	0.5832	1.0584	0.8641	1.2964
SOFA	0.1304	0.1616	0.4198	1.1393	0.8299	1.5639
MPO	0.0808	0.0402	0.0446	1.0841	1.0020	1.1730
NE	0.0526	0.0248	0.0339	1.0540	1.0040	1.1065

### Clinical features of patients with sepsis with different neutrophil granule protein levels

Based on the cutoff level of neutrophil granule proteins in ROC curve analysis, all the patients were divided into a low neutrophil granule protein group and a high neutrophil granule protein group, with high levels of both MPO and NE. **Table 4** shows the clinical characteristics of patients in the different neutrophil granule protein groups. Age, sex, and infection site were in agreement between the groups. Gram-negative bacilli (73.3% vs. 45.2%, *p* = 0.0374) and fungi (55.0% vs. 12.9%, *p* = 0.0402) were more prevalent in the high neutrophil granule protein level group. The mortality (23.3% vs. 3.2%, *p* = 0.0261), ICU stay time (6.0 vs. 3.0, *p* = 0.0451), APACHE II score (18.4 vs. 13.2, *p* = 0.0030), and SOFA score (9.8 vs. 6.5, *p* = 0.0018) were significantly greater in the high neutrophil granule protein level group than in the low neutrophil granule protein level group. Both the need for mechanical ventilation (96.7% vs. 74.2%, *p* = 0.0261) and the need for renal replacement (33.3% vs. 9.7%, *p* = 0.0311) were significantly greater in the high neutrophil granule protein level group. The levels of inflammatory biomarkers, such as CRP (186.4 vs. 121.2, *p* = 0.0160), IL-6 (276.0 vs. 46.9, *p* = 0.0094), and IL-8 (64.0 vs. 35.7, *p* = 0.0410) were significantly different in the groups with different neutrophil granule protein levels. Lower lymphocyte counts and subset cell counts were seen in patients with high neutrophil granule protein levels. Overall, patients with sepsis with high neutrophil granule protein levels had worse outcomes and experienced longer ICU stay times and more severe organ dysfunction.

**Table 4:** The clinical characteristics of patients with different neutrophil granule proteins level.

Characteristics	Low neutrophil granule proteins, n = 31	High neutrophil granule proteins, n = 30	p
Age, years	58.2 (41.5 – 74.9)	64.0 (48.9 – 79.2)	0.1656
Sex, n (%) Male	19 (61.3)	20 (66.7)	0.7911
Infection site, n (%)			
Bloodstream	5 (16.1)	4 (13.3)	0.9999
Lung	11 (35.5)	15 (50.0)	0.3056
Abdominal cavity	11 (35.5)	16 (53.3)	0.2016
Soft tissue	3 (9.7)	2 (6.7)	0.9999
Pathogen, n (%)			
Gram-negative bacilus	14 (45.2)	22 (73.3)	0.0374^*^
Gram-positive coccus	9 (29.0)	16 (53.3)	0.0707
Gram-positive bacilus	2 (6.5)	2 (6.7)	0.9999
Fungus	4 (12.9)	11 (55.0)	0.0402^*^
28-day mortality, n (%)	1 (3.2)	7 (23.3)	0.0261^*^
ICU stay time, days	3.0 (2.0 – 8.5)	6.0 (3.0 – 10.3)	0.0451^*^
APACHE II	13.2 (6.8 – 19.6)	18.4 (11.9 – 24.9)	0.0030^**^
SOFA	6.5 (3.1 – 10.0)	9.8 (5.7 – 13.9)	0.0018^**^
MV, n (%)	23 (74.2)	29 (96.7)	0.0261^*^
CRRT, n (%)	3 (9.7)	10 (33.3)	0.0311^*^
PCT, ng/mL	0.6 (0.3 – 2.7)	6.8 (1.3 – 11.0)	0.1588
CRP, mg/L	121.2 (35.9 – 206.5)	186.4 (71.7 -301.0)	0.0160^*^
WBC, *10^9/L	11.9 (8.4 – 19.3)	12.8 (8.5 – 14.9)	0.5554
LY#, *10^6/L	1249 (598 – 1900)	503 (276 – 484)	<0.0001^****^
CD4^+^ T#, *10^6/L	443 (230 – 655)	192 (58 – 171)	<0.0001^****^
CD8^+^ T#, *10^6/L	320 (40 – 599)	125 (41 – 126)	0.0006^***^
IL-6, pg/mL	46.9 (17.5 – 113.0)	276.0 (65.1 – 1000.0)	0.0094^**^
IL-8, pg/mL	35.7 (29.0 – 60.1)	64.0 (38.0 – 145.0)	0.0410^*^
IL-10, pg/mL	7.4 (5.0 – 12.8)	11.3 (5.2 – 29.6)	0.2921
TNFα, pg/mL	9.5 (2.5 – 18.2)	12.1 (2.5 – 18.9)	0.7503

*p<0.05; **p<0.01; ***p<0.001; ****p<0.000.

### Levels of biomarkers of pyroptosis in CD4^+^ T cells were associated with neutrophil granule protein levels in patients with sepsis

Biomarkers of pyroptosis in CD4^+^ T cells were evaluated in groups with different neutrophil granule protein levels. The level of NLRP3 [78.4 (65.9–90.9) vs. 31.3 (6.2–56.3), *p* < 0.0001^****^] was significantly higher in the high neutrophil granule protein level group, and the levels of the components of its subsequent pathway, Caspase-1 [56.2 (36.0–76.4) vs. 19.4 (1.4–37.5), *p* < 0.0001^****^] and GSDMD [26.5 (17.4–35.6) vs. 7.9 (0.9–14.9), *p* < 0 <.0001^****^], were higher in the high neutrophil granule protein level group (shown in **Figure 1**, **Table 5**).

**Table 5:** Levels of biomarkers of pyroptosis expressed on CD4+ T cells in groups with different neutrophil granule proteins levels.

Characteristics	Low neutrophil granule proteins, n = 31	High neutrophil granule proteins, n = 30	p
CD4^+^ T#, *10^6/L	443 (230 – 655)	192 (58 – 171)	<0.0001^****^
CD4^+^NLRP3^+^, %	31.3 (6.2 – 56.3)	78.4 (65.9 – 90.9)	<0.0001^****^
CD4^+^Caspase-1^+^, %	19.4 (1.4 – 37.5)	56.5 (36.6 – 76.5)	<0.0001^****^
CD4^+^GSDMD^+^, %	7.9 (0.9 – 14.9)	26.5 (17.4 – 35.6)	<0.0001^****^

*p<0.05; ****p<0.0001.

### Relationships among neutrophil granule proteins, CD4^+^ T cells, and pyroptosis

**Figure 5:**
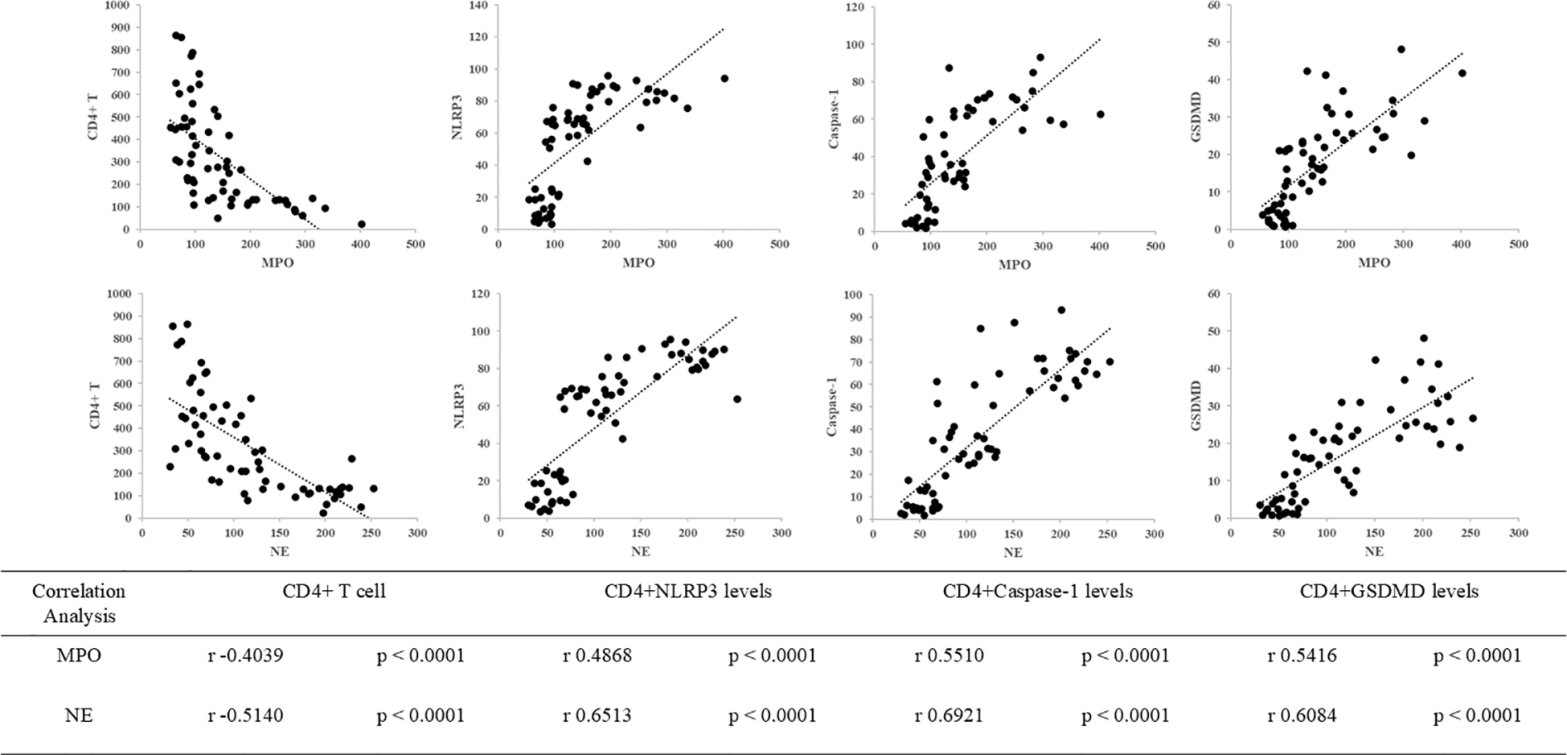
Correlations between neutrophil granule protein level (including MPO and NE), CD4^+^ T cells, and pyroptosis biomarkers (including NLRP3, Caspase-1, and GSDMD) in patients with sepsis. The correlation between MPO levels and CD4^+^ T cells (*r* = −0.4039, *p* < 0.0001), MPO levels and CD4^+^NLRP3 levels (*r* = 0.4868, *p* < 0.0001), MPO levels and CD4^+^Caspase-1 levels (*r* = 0.5510, *p* < 0.0001), and MPO levels and CD4^+^GSDMD levels (*r* = 0.5416, *p* < 0.0001). The correlation between NE levels and CD4^+^ T cells (*r* = −0.5140, *p* < 0.0001), NE levels and CD4^+^NLRP3 levels (*r* = 0.6513, *p* < 0.0001), NE levels and CD4^+^Caspase-1 levels (*r* = 0.6921, *p* = 0.0002), and NE levels and CD4^+^GSDMD levels (*r* = 0.6084, *p* < 0.0001).

We hypothesized that neutrophil granule proteins, CD4^+^ T cells, and pyroptosis interact closely in the mechanism underlying SAL. Consistent with this hypothesis, Pearson’s correlation analysis revealed significant correlations between MPO levels and CD4^+^ T cells (*r* = −0.4039, *p* < 0.0001), CD4^+^NLRP3 levels (*r* = 0.4868, *p* < 0.0001), CD4^+^Caspase-1 levels (*r* = 0.5510, *p* < 0.0001), and CD4^+^GSDMD levels (*r* = 0.5416, *p* < 0.0001), as well as between NE levels and CD4^+^ T cells (*r* = −0.5140, *p* < 0.0001), CD4^+^NLRP3 levels (*r* = 0.6513, *p* < 0.0001), CD4^+^Caspase-1 levels (*r* = 0.6921, *p* = 0.0002), and CD4^+^GSDMD levels (*r* = 0.6084, *p* < 0.0001) (**Figure 5**). A significant relationship between neutrophil granule proteins and the pyroptosis pathway of CD4^+^ T cells was revealed.

## Discussion

Our study first evaluated the role of plasma neutrophil granule protein level in patients with SAL. To explore the relationship between neutrophil granule proteins and SAL, we evaluated the levels of plasma neutrophil granule proteins within 24 h after admission. We found that higher plasma neutrophil granule protein levels showed close relationship with the incidence of lymphopenia in patients with sepsis. Moreover, patients with sepsis with high neutrophil granule protein levels had lower CD4^+^ T lymphocyte counts and higher levels of biomarkers of pyroptosis. The significant correlation between neutrophil granule proteins and CD4^+^ T-cell counts and levels of biomarkers of pyroptosis revealed that plasma neutrophil granule proteins may play a role in SAL through the pyroptosis pathway of CD4^+^ T cells.

Immunosuppressive changes, often implicated in increased susceptibility to secondary infections (16, 17), are a main cause of overall mortality of patients with sepsis (18). The immune system is composed of the innate and the adaptive immune system. Research over the last decade has documented neutrophil heterogeneity and functional versatility far beyond their antimicrobial function (19). Emerging evidence indicates that neutrophils utilize granule proteins to interact with innate and adaptive immune cells and regulate the inflammatory response. Our study explored that patients with SAL had higher plasma neutrophil granule protein levels than controls did. Neutrophil granule proteins were independently associated with lymphopenia risk (MPO: OR = 1.0841, 95% CI, 1.0020–1.1730, *p* = 0.0446; NE: OR = 1.0540, 95% CI, 1.0040–1.1065, *p* = 0.0339). The area under the ROC curve in predicting SAL occurrence for MPO was 0.939 (95% CI, 0.846–0.984; *p* < 0.0001), whereas that for NE was 0.950 (95% CI, 0.862–0.989; *p* < 0.0001). Overall, patients with sepsis with high neutrophil granule protein levels had longer ICU stays and higher mortality, more severe organ dysfunction, and frequent need for organ support. Our study firstly explored the effects of neutrophil granule proteins in patients with sepsis with lymphopenia.

Different cell types and characteristics were involved in immunosuppression in patients with sepsis. A major reason for immunosuppression in sepsis is the depletion of immune cells (20, 21). Notably, the abnormal quantity and function of CD4^+^ T cells cannot be ignored in the development and progression of immunosuppression (22). Previous research has shown that NETs may interact with adaptive immune cells through different pathways (23, 24). In our study, patients with high neutrophil granule proteins had significantly fewer CD4^+^ T cells [192 (58–171) vs. 443 (230–655), *p* < 0 <.0001]. Neutrophil granule proteins were closely related to the CD4^+^ T-cell count (MPO vs. CD4^+^ T-cell count, *r* = −0.4039, *p* < 0.0001; NE vs. CD4^+^ T-cell count, *r* = −0.5140, *p* < 0.0001). In addition to traditional signal transduction between the innate and adaptive immune system (21, 25), the connections between neutrophil granule proteins and T lymphocytes deserve further exploration.

As a type of programmed cell death discovered in recent years, pyroptosis of immune cell in sepsis is complicated (26). A protective role in sepsis was initially thought with the downregulation of inflammation, but an increasing number of studies have demonstrated the disadvantages of excessive pyroptosis (27, 28). Observational study findings have suggested that the NLRP3 inflammasome may drive post-septic immunosuppression (29, 30). Our previous study verified that canonical pyroptosis is an important mechanism underlying CD4^+^ T-cell lymphocytopenia during sepsis (8, 31). In this study, biomarkers of CD4^+^ T-cell pyroptosis were evaluated in groups with different neutrophil granule protein levels. Levels of NLRP3 [78.4 (65.9–90.9) vs. 31.3 (6.2–56.3), *p* < 0.0001], Caspase-1 [56.2 (36.0–76.4) vs. 19.4 (1.4–37.5), *p* < 0.0001], and GSDMD [26.5 (17.4–35.6) vs. 7.9 (0.9–14.9), *p* < 0 <.0001] were significantly higher in the high neutrophil granule protein level group. Neutrophil granule proteins are closely related to CD4^+^ T-cell pyroptosis. We hypothesize that neutrophil granule proteins may participate in SAL through CD4^+^ T-cell pyroptosis.

The impact of neutrophil granule proteins on immune responses is largely implicated in APC T-cell immunity. MPO can modulate immune responses by either CD4^+^ T -cell activation or dendritic cell suppression, and elastase can potentially promote Th17 response but simultaneously induces DC production of TGF-β for suppressing T-cell proliferation (32, 33). Our study first explored crosstalk between neutrophil granule proteins and the pyroptosis pathway of CD4^+^ T cells. Neutrophil granule proteins have been identified as important modulators of neutrophil trafficking, reverse transendothelial migration, phagocytosis, neutrophil life span, NET formation, efferocytosis, cytokine activity, and autoimmunity (5). Based on recent studies, NETs may interact with pyroptosis signal pathway through different approaches. Increased NETs may induce expression of pyroptosis-related proteins and promote NLRP3 inflammasome activation (9, 10). The STING/IRE1α signaling pathway or the NF-κB/caspase 3/GSDME axis may contribute to the process (34, 35). However, the complex regulatory mechanisms in sepsis are still worth exploring.

There are still several limitations in our study. As a single-center study, it may limit the generalizability of the results. Relatively small sample and complex confounders may lower the credibility of the results. A larger sample size or larger multicenter studies should be considered. Second, although the correlation between neutrophil granule proteins and CD4^+^ T-cell pyroptosis was identified in this research, the causal relationship still requires further basic studies. More than that, immunosuppression is a complex status that may change during different courses of disease. In our study, we only collected blood samples within 24 h after being admitted, and a dynamic analysis of neutrophil granule proteins may be worthy of further study. Finally, neutrophil granule protein detections still have several limitations because plasma neutrophil granule proteins can be induced by different mechanisms like neutrophil degranulation, necroptosis, pyroptosis, or NETosis. Further animal experiments are still needed to explore detailed mechanisms.

## Conclusion

Increased levels of neutrophil granule proteins were significantly associated with SAL incidence, and a significant relationship between neutrophil granule proteins and the pyroptosis pathway of CD4^+^ T cells was revealed.

## Data Availability

The datasets presented in this study can be found in online repositories. The names of the repository/repositories and accession number(s) can be found in the article/supplementary material.

## References

[1] Singer M Deutschman CS Seymour CW Shankar-Hari M Annane D Bauer M. The third international consensus definitions for sepsis and septic shock (Sepsis-3). JAMA. (2016) 315:801–10. doi: 10.1001/jama.2016.0287 PMC4968574 26903338

[2] van der Poll T Shankar-Hari M Wiersinga WJ. The immunology of sepsis. Immunity. (2021) 54:2450–64. doi: 10.1016/j.immuni.2021.10.012 34758337

[3] Tsai CY Hsieh SC Liu CW Lu CS Wu CH Liao HT. Cross-Talk among Polymorphonuclear Neutrophils, Immune, and Non-Immune Cells via Released Cytokines, Granule Proteins, Microvesicles, and Neutrophil Extracellular Trap Formation: A Novel Concept of Biology and Pathobiology for Neutrophils. Int J Mol Sci. (2021) 22(6):3119. doi: 10.3390/ijms22063119 33803773 PMC8003289

[4] Brinkmann V Reichard U Goosmann C Fauler B Uhlemann Y Weiss DS. Neutrophil extracellular traps kill bacteria. Science. (2004) 303:1532–5. doi: 10.1126/science.1092385 15001782

[5] Othman A Sekheri M Filep JG. Roles of neutrophil granule proteins in orchestrating inflammation and immunity. FEBS J. (2022) 289:3932–53. doi: 10.1111/febs.v289.14 PMC9546106 33683814

[6] Vigneron C Py BF Monneret G Venet F. The double sides of NLRP3 inflammasome activation in sepsis. Clin Sci (Lond). (2023) 137:333–51. doi: 10.1042/CS20220556 36856019

[7] Liu D Huang SY Sun JH Zhang HC Cai QL Gao C. Sepsis-induced immunosuppression: mechanisms, diagnosis and current treatment options. Mil Med Res. (2022) 9:56. doi: 10.1186/s40779-022-00422-y 36209190 PMC9547753

[8] Guo R Zhao G Bai G Chen J Han W Cui N. Depletion of mTOR ameliorates CD(4)(+) T cell pyroptosis by promoting autophagy activity in septic mice. Int Immunopharmacol. (2023) 124:110964. doi: 10.1016/j.intimp.2023.110964 37738689

[9] Cui Y Yang Y Tao W Peng W Luo D Zhao N. Neutrophil extracellular traps induce alveolar macrophage pyroptosis by regulating NLRP3 deubiquitination, aggravating the development of septic lung injury. J Inflammation Res. (2023) 16:861–77. doi: 10.2147/JIR.S366436 PMC9983334 36876152

[10] Liu C Zhou Y Tu Q Yao L Li J Yang Z. Alpha-linolenic acid pretreatment alleviates NETs-induced alveolar macrophage pyroptosis by inhibiting pyrin inflammasome activation in a mouse model of sepsis-induced ALI/ARDS. Front Immunol. (2023) 14:1146612. doi: 10.3389/fimmu.2023.1146612 37051243 PMC10083395

[11] Pei F Yao RQ Ren C Bahrami S Billiar TR Chaudry IH. Expert consensus on the monitoring and treatment of sepsis-induced immunosuppression. Mil Med Res. (2022) 9:74. doi: 10.1186/s40779-022-00430-y 36567402 PMC9790819

[12] Borregaard N Cowland JB. Granules of the human neutrophilic polymorphonuclear leukocyte. Blood. (1997) 89:3503–21. doi: 10.1182/blood.V89.10.3503 9160655

[13] Broz P Dixit VM. Inflammasomes: mechanism of assembly, regulation and signalling. Nat Rev Immunol. (2016) 16:407–20. doi: 10.1038/nri.2016.58 27291964

[14] Moltrasio C Romagnuolo M Marzano AV. NLRP3 inflammasome and NLRP3-related autoinflammatory diseases: From cryopyrin function to targeted therapies. Front Immunol. (2022) 13:1007705. doi: 10.3389/fimmu.2022.1007705 36275641 PMC9583146

[15] Shi J Zhao Y Wang K Shi X Wang Y Huang H. Cleavage of GSDMD by inflammatory caspases determines pyroptotic cell death. Nature. (2015) 526:660–5. doi: 10.1038/nature15514 26375003

[16] Ong DSY Bonten MJM Spitoni C Verduyn Lunel FM Frencken JF Horn J. Epidemiology of multiple herpes viremia in previously immunocompetent patients with septic shock. Clin Infect Dis. (2017) 64:1204–10. doi: 10.1093/cid/cix120 28158551

[17] Otto GP Sossdorf M Claus RA Rödel J Menge K Reinhart K. The late phase of sepsis is characterized by an increased microbiological burden and death rate. Crit Care. (2011) 15:R183. doi: 10.1186/cc10332 21798063 PMC3387626

[18] Rudd KE Johnson SC Agesa KM Shackelford KA Tsoi D Kievlan DR. Global, regional, and national sepsis incidence and mortality, 1990-2017: analysis for the Global Burden of Disease Study. Lancet. (2020) 395:200–11. doi: 10.1016/S0140-6736(19)32989-7 PMC6970225 31954465

[19] Cassatella MA Östberg NK Tamassia N Soehnlein O. Biological roles of neutrophil-derived granule proteins and cytokines. Trends Immunol. (2019) 40:648–64. doi: 10.1016/j.it.2019.05.003 31155315

[20] Hotchkiss RS Monneret G Payen D. Sepsis-induced immunosuppression: from cellular dysfunctions to immunotherapy. Nat Rev Immunol. (2013) 13:862–74. doi: 10.1038/nri3552 PMC4077177 24232462

[21] van der Poll T van de Veerdonk FL Scicluna BP Netea MG. The immunopathology of sepsis and potential therapeutic targets. Nat Rev Immunol. (2017) 17:407–20. doi: 10.1038/nri.2017.36 28436424

[22] Martin MD Badovinac VP Griffith TS. CD4 T cell responses and the sepsis-induced immunoparalysis state. Front Immunol. (2020) 11:1364. doi: 10.3389/fimmu.2020.01364 32733454 PMC7358556

[23] Melero I Villalba-Esparza M Recalde-Zamacona B Jiménez-Sánchez D Teijeira Á Argueta A. Neutrophil extracellular traps, local IL-8 expression, and cytotoxic T-lymphocyte response in the lungs of patients with fatal COVID-19. Chest. (2022) 162:1006–16. doi: 10.1016/j.chest.2022.06.007 PMC9197577 35714708

[24] Wilson AS Randall KL Pettitt JA Ellyard JI Blumenthal A Enders A. Neutrophil extracellular traps and their histones promote Th17 cell differentiation directly via TLR2. Nat Commun. (2022) 13:528. doi: 10.1038/s41467-022-28172-4 35082281 PMC8792063

[25] Tillack K Breiden P Martin R Sospedra M. T lymphocyte priming by neutrophil extracellular traps links innate and adaptive immune responses. J Immunol. (2012) 188:3150–9. doi: 10.4049/jimmunol.1103414 22351936

[26] Zheng X Chen W Gong F Chen Y Chen E. The role and mechanism of pyroptosis and potential therapeutic targets in sepsis: A review. Front Immunol. (2021) 12:711939. doi: 10.3389/fimmu.2021.711939 34305952 PMC8293747

[27] Gao YL Zhai JH Chai YF. Recent advances in the molecular mechanisms underlying pyroptosis in sepsis. Mediators Inflamm. (2018) 2018:5823823. doi: 10.1155/2018/5823823 29706799 PMC5863298

[28] Jorgensen I Miao EA. Pyroptotic cell death defends against intracellular pathogens. Immunol Rev. (2015) 265:130–42. doi: 10.1111/imr.2015.265.issue-1 PMC4400865 25879289

[29] Wang YC Liu QX Liu T Xu XE Gao W Bai XJ. Caspase-1-dependent pyroptosis of peripheral blood mononuclear cells predicts the development of sepsis in severe trauma patients: A prospective observational study. Med (Baltimore). (2018) 97:e9859. doi: 10.1097/MD.0000000000009859 PMC5841964 29465571

[30] Roth S Cao J Singh V Tiedt S Hundeshagen G Li T. Post-injury immunosuppression and secondary infections are caused by an AIM2 inflammasome-driven signaling cascade. Immunity. (2021) 54:648–59.e8. doi: 10.1016/j.immuni.2021.02.004 33667383

[31] Zhao G Xie Y Lei X Guo R Cui N. mTOR aggravated CD4(+) T cell pyroptosis by regulating the PPARγ-Nrf2 pathway in sepsis. Int Immunopharmacol. (2024) 140:112822. doi: 10.1016/j.intimp.2024.112822 39096877

[32] Gan PY Holdsworth SR Kitching AR Ooi JD. Myeloperoxidase (MPO)-specific CD4+ T cells contribute to MPO-anti-neutrophil cytoplasmic antibody (ANCA) associated glomerulonephritis. Cell Immunol. (2013) 282:21–7. doi: 10.1016/j.cellimm.2013.04.007 23665205

[33] Souwer Y Groot Kormelink T Taanman-Kueter EW Muller FJ van Capel TMM Varga DV. Human T(H)17 cell development requires processing of dendritic cell-derived CXCL8 by neutrophil elastase. J Allergy Clin Immunol. (2018) 141:2286–9.e5. doi: 10.1016/j.jaci.2018.01.003 29391256

[34] Cao Y Shi M Liu L Zuo Y Jia H Min X. Inhibition of neutrophil extracellular trap formation attenuates NLRP1-dependent neuronal pyroptosis via STING/IRE1α pathway after traumatic brain injury in mice. Front Immunol. (2023) 14:1125759. doi: 10.3389/fimmu.2023.1125759 37143681 PMC10152368

[35] Mao J Tan M Li J Liu C Hao J Zheng J. Neutrophil extracellular traps induce pyroptosis of rheumatoid arthritis fibroblast-like synoviocytes via the NF-κB/Caspase 3/GSDME pathway. Inflammation. (2024) 47:921–38. doi: 10.1007/s10753-023-01951-x 38133702

